# Shedding light on amphipod behaviour: Baseline locomotion behaviour for laboratory ecotoxicology experiments

**DOI:** 10.1371/journal.pone.0357763

**Published:** 2026-10-07

**Authors:** Susanne Vogeler, Matthew O. Parker, Samuel C. Robson, Alex T. Ford

**Affiliations:** 1 Institute of Marine Sciences, University of Portsmouth, Portsmouth, United Kingdom; 2 Faculty of Life and Health Sciences, University of Exeter, Exeter, United Kingdom; 3 Surrey Sleep Research Centre, School of Bioscience, University of Surrey, Guildford, United Kingdom; 4 Institute of Life Sciences and Healthcare, University of Portsmouth, Portsmouth, United Kingdom; King Abdulaziz University, SAUDI ARABIA

## Abstract

Behavioural endpoints offer a sensitive, non-invasive alternative to traditional mortality assays. Yet, they remain underutilized in regulatory frameworks due to a lack of standardised methodologies and baseline behavioural data. This study investigates the baseline locomotion of the marine amphipod *Marinogammarus marinus* to external light triggers to support standardised laboratory behavioural assays. Using an automated observation chamber, here we tracked swimming behaviour in response to light stimuli, quantifying locomotion as distance travelled. Groups of eight amphipods were tested in large crystalizing dishes over four days in an 8-minute trial involving two light exposures. A total of 60 individuals – males, females, and brooding females – were assessed. Light consistently triggered activity, with males showing strong negative phototaxis and brooding females the weakest. A startle response was observed within 3–5 seconds of initial light exposure, though responses diminished with repetition, suggesting potential overstimulation or habituation. Inter-individual and day-to-day variability highlighted the need for repeated measures. Additional trials with 32 males evaluated the effects of experimental conditions. Wall-hugging (thigmotaxis) appeared to be an escape response, not anxiety-related. Notable decreases in dissolved oxygen and pH within 24 hours were attributed to the amphipods’ presence rather than activity, while salinity fluctuations due to evaporation did not visibly affect behaviour. Differences between male groups from the two separate experiments suggest behavioural shifts due to seasonal variation. This study establishes baseline behavioural data for *M. marinus* and demonstrates the value of automated tracking systems in behavioural assays in laboratory settings. It emphasizes the need to account for sex, size, and temporal variation in assay design and supports integrating behavioural endpoints into regulatory protocols.

## 1. Introduction

The implementation of standardised behavioural assays below sub-lethal concentrations for understanding and regulating ecotoxicological impact is still a slow process [1], even though behaviour is an ideal endpoint as it is generally a more sensitive indicator of adverse effects in the environment than conventional acute or chronic mortality studies [1–5]. Behaviour is classified as the observable reactions and responses of an organism to physiological and environmental factors [6] and is therefore closely linked to ecological processes including individual fitness, population dynamics, species interactions, and ecosystem health [1,7,8]. Behavioural observations are non-invasive and enable repeated and long-term measurements. Despite increasing research on behavioural responses in laboratory and field ecotoxicology over recent decades [5,8,9], such endpoints are still rarely incorporated into regulatory chemical risk assessment guidelines due to limited standardised and validated test methods [1,9].

Where behavioural tests are implemented, they typically serve as supportive rather than stand-alone endpoints [10]. To advance standardised methods, rigorous baseline data are required regarding species selection, behavioural endpoints, and reproducible experimental designs [9,10]. Species choice and behavioural endpoints should reflect their suitability to the scientific goal or testing parameters. With behaviours varying greatly between individuals, testing a larger number of animals is vital to get a more accurate assessment of the overall behaviour of a species population. High-throughput system units, commercially available (DanioVision – Noldus [11], ToxmateLab & ZebraBox – Viewpoint, Zantiks systems – Zantiks, DaphniaToximeter II – bbe moldaenke) or custom-made (Lab-on-Chips [12–14]) for testing animals including meio- & macrofauna have been developed over the past decade. A particular challenge exists for studies on aquatic species, given issues in recording behaviours and regulating experimental conditions in water. Integrated video-based monitoring systems that regulate environmental parameters such as light, temperature, noise, water flow, and chemical exposure provide powerful tools for quantifying behavioural responses [15,16]. Establishing baseline behaviour, identifying reliable behavioural triggers, and accounting for individual variability are therefore critical steps toward standardised test development.

Aquatic amphipods are one of the most ecologically and species-diverse Order of crustaceans and are widely distributed across freshwater, estuarine, and marine habitats [17,18]. They play crucial roles in aquatic food webs, as grazers, shredders, predators or as food sources for bigger predators such as fish and birds [18–20], and are widely recognized as effective indicators of environmental impacts [21–26]. Their distinct and quantifiable behaviours are closely linked to fitness, population dynamics, and ecosystem functioning [18,27–29].

Swimming is a primary mode of locomotion in many aquatic species and can be readily quantified using video-based systems. Many amphipods exhibit negative phototaxis [30–33], likely a predator avoidance adaptation to swim away from light sources to avoid being seen by predators [34–36]. Thus, light can be used as a trigger to induce swimming behaviour. As such, locomotion represents a sensitive and controllable endpoint. In addition, amphipods are sexually dimorphic and display characteristic reproductive behaviours, including precopulatory mate guarding (amplexus), during which males clasp females for extended periods [37]. Swimming, amplexus formation, foraging, shelter-seeking as well as respiration have all been used to assess sub-lethal pollutant effects [14,23,31,38–48]. Despite this extensive evidence of the effectiveness of amphipods in behavioural assays for ecotoxicology, few regulatory guidelines (OECD 321; ICES TIMES No. 28 and No. 41) include amphipods, and none incorporate behavioural endpoints.

A key limitation to broader regulatory implementation is the lack of systematic baseline characterisation and methodological standardisation. Reliable behavioural assays require reproducible quantification across laboratories and clear definition of experimental parameters, including arena dimensions, light regimes, animal density, exposure duration, water maintenance, sex and developmental stage selection, and sampling time points. Variability in these parameters can substantially influence behavioural measurements and limiting cross-study comparability.

The marine intertidal amphipod species *Marinogammarus marinus* (formally *Echinogammarus marinus*) is abundant along European coasts [49,50], has low rearing requirements, and is tolerant to fluctuations in light, salinity, and temperature [51,52]. These characteristics are comparable to those of freshwater amphipod species commonly used in ecotoxicological testing, such as *Gammarus* spp. As a marine intertidal species, however, *M. marinus* broadens the range of taxa available for behavioural ecotoxicology in coastal environments. Furthermore, it can be readily collected in large numbers, maintained under laboratory conditions with minimal husbandry requirements, and exhibits robust behavioural responses to environmental stimuli, making it a practical candidate for high-throughput behavioural screening. Previous studies have demonstrated that this species, as well as other amphipods, responds to short light stimuli with increased locomotion [31,42,43,47]. Sex-specific differences in light-induced locomotive behaviour have been reported in *M. marinus* [39] and assessment container shape and size have been shown to influence behavioural outputs [40]. Moreover, pharmaceutical compounds and environmental pollutants have been detected to alter amphipod locomotion using similar behavioural paradigms [30,41,42,47,48]. Together, these studies highlight both the sensitivity of amphipod swimming behaviour as an ecotoxicological endpoint and the susceptibility of behavioural measurements to experimental design variables.

Although sex-specific responses and certain methodological variables have been investigated, these variables have rarely been examined concurrently within a single, standardised, high-throughput framework. In particular, the locomotive behaviour of brooding females carrying developing juveniles has not been explicitly characterised, and behavioural variability under repeated daily automated testing remains insufficiently described. Such integrative baseline data are essential for refining experimental design and improving reproducibility.

In the present study, we developed and evaluated a simplified, automated, high-throughput behavioural assay using the Zantiks LT system (Zantiks Ltd.), an integrated infrared video-tracking platform previously applied in vertebrate and *Drosophila* research [53–55], but not yet systematically validated for amphipod locomotion studies. *M. marinus* exhibits strong negative phototaxis [30,31], making light an ideal inducer of locomotion. Sudden changes in light intensity can also trigger rapid escape or startle responses, which are acute involuntary reactions to abrupt stimuli such as light onset [56]. These responses are considered conserved innate behaviours across crustaceans [57] and have increasingly been used as sensitive endpoints in amphipod ecotoxicological studies [43,48]. Using a top-down tracking approach, we quantified light-induced swimming behaviour in individual animals housed in standardised crystallising dishes, enabling the assessment of up to 60 individuals within 90 minutes per day across four consecutive days.

Locomotion was analysed across sex categories, including males, non-brooding females, and, for the first time, brooding females, allowing assessment of reproductive status as a potential behavioural modifier. Because amphipods carry developing offspring within a marsupium until release, brood number and juvenile body length were also quantified to explore potential associations between brood characteristics and female locomotive activity. Brood number and juvenile body length were quantified to explore associations between brood presence and female locomotive activity. We further examined body length effects, repeated daily variability, thigmotaxis behaviour, and water condition changes throughout the experiment.

By systematically characterising sex-, size-, and reproductive status-specific baseline swimming behaviour over a four-day time course using an automated high-throughput platform, this study provides foundational data for improving reproducibility and supporting the future standardisation of behavioural assays with amphipods.

## 2. Methods

### 2.1 Species collection and husbandry

Male and female *M. marinus* individuals were collected from Lock Lake, Langstone Harbour, Portsmouth, UK (coordinates 50°47’22.8“N, 1°02’33.2”W) at low tide. Specimens were acclimatised in the laboratory for two weeks in static aerated artificial seawater (ASW) prepared with Classic Sea Salt (Tropic Marin) in reverse-osmosis water (RO-water) at 32.0 ± 0.5 ppt, pH 8.20 ± 0.06, at 10 ± 1 °C under a 24-hour dark photoperiod (approximately 3x 30 animals in 5L ASW, water changed twice a week). The photoperiod and acclimation time were chosen to eliminate potential alterations in behaviour due to circadian and circatidal rhythms [58]. During acclimation, animals were fed a mixture of *Fucus vesiculosus* and *Ascophyllum nodosum*, which also provided substrate for the marine amphipods. Animals of different sexes for behavioural assessment were collected during winter (January 2022, mean water temperature 9.4 °C), while male-only specimens for behavioural assessment with experimental stressors were collected during the summer months (July 2022, mean water temperature 18.5°C) [59].

### 2.2 Behavioural assessment – different sexes and reproductive conditions

Prior to behavioural assessment, animals collected in January were separated into three groups, referred to as “sex groups”: males, females (non-brooding; no eggs or juveniles visible in the marsupium), and brooding females (juveniles visible in the marsupium), using a stereoscopic microscope. Males were differentiated from females by the presence of larger gnathopods (front claws) and the absence of oostegites (brood plates). Animals with visible injuries such as missing limbs (legs, antennae) or visible parasites were excluded. Twenty individuals per group (males, females, and brooding females) were randomly selected. Each individual was placed in a borosilicate glass crystallising dish (90 mm diameter) with 100 ml ASW (32.1 ppt, pH 8.22) loosely covered with plastic lids to allow for gas exchange. Animals were incubated in the dishes for 96 h at 10 °C in the dark without aeration or feeding in a cooled incubator (PHCbi) and assessed daily over the 96h incubation period for selected behavioural endpoints. Before each daily assessment, moulting events and mortality were recorded. Brooding females were checked daily for released juveniles, which were counted and removed. Juveniles were anaesthetised with 0.01% clove oil in ASW (100% clove oil, Aroma Labs, France) and photographed. The 20% stock solution of clove oil was prepared in 70% ethanol. At the end of the experiment, adult amphipods were anaesthetised with 0.025% clove oil in ASW for 5 minutes. Sex and breeding status were reassessed. The marsupia of females still containing juveniles were flushed with ASW using a 1 ml pipette to remove remaining juveniles. All adults and juveniles were photographed using a Leica S8AP0 stereo microscope equipped with a Leica MC120 HD camera. Body length (from the insertion of the first antenna to telson) was measured using ImageJ v1.54f. Amphipods were terminated after another anaesthesia with 0.025% clove oil and then frozen at −20°C. After reassessment of the sex and reproductive stage at the end of the experiment, two females were moved to brooding female group due to inaccurate initial classification as non-brooding, and one was excluded (contained eggs). This left 20 replicates for males, 17 replicates for females, and 22 replicates for brooding females. No moults or mortality has been observed during the experiment.

Behaviour was assessed using the automated Zantiks LT system (Zantiks Ltd, Cambridge, UK). Locomotion, measured as the distance travelled (in mm) per second in a confined arena, was chosen as behavioural endpoint (Fig 1). Locomotion was assessed in the crystallising dishes at room temperature in the Zantiks LT unit (unit not temperature controlled). The unit was placed upside down, with dishes positioned on the overhead light plate rather than the screen in the experimental chamber. The chamber was light-sealed to prevent external light exposure. Preliminary tests showed that tracking amphipods placed on the overhead light plate was superior to placing them on the screen. Exposure to light was used as a stimulus to induce locomotion behaviour. Amphipods are negatively phototactic (prefer dark environments); therefore, short light exposure induces swimming behaviour as previously shown [30,31]. Horizontal locomotion of each animal was tracked over an 8-min period using alternating 2-min dark-light-dark-light conditions with the built-in infrared camera and Zantiks software (Pre-release, v2022-01–11). Eight animals were assessed per run, with eight runs conducted to test all 60 animals. To reduce technical error, animals were randomly assigned positions within the unit for each run, combining 2–4 replicates of each sex group per run. No correlations between the position of the dish in the observation chamber and distance travelled per animal have been detected (S1 File). White light (297.5 ± 5.7 lux) was emitted from the overhead light plate. Prior to tracking, a 60-sec auto-reference period and a 2-min dark pre-trial were used to ensure tracking accuracy and allow animals to acclimate following handling. Preliminary tests showed that longer acclimation did not further reduce locomotive activity, and amphipods typically remain inactive in darkness due to negative phototaxis. Locomotion assessments were performed at 24 h, 48 h, 72 h, and 96 h, at the same time each day. Animals in their crystallising dishes were removed from the incubator shortly before assessment and returned immediately afterward to minimise exposure to room temperature and light.

**Fig 1:**
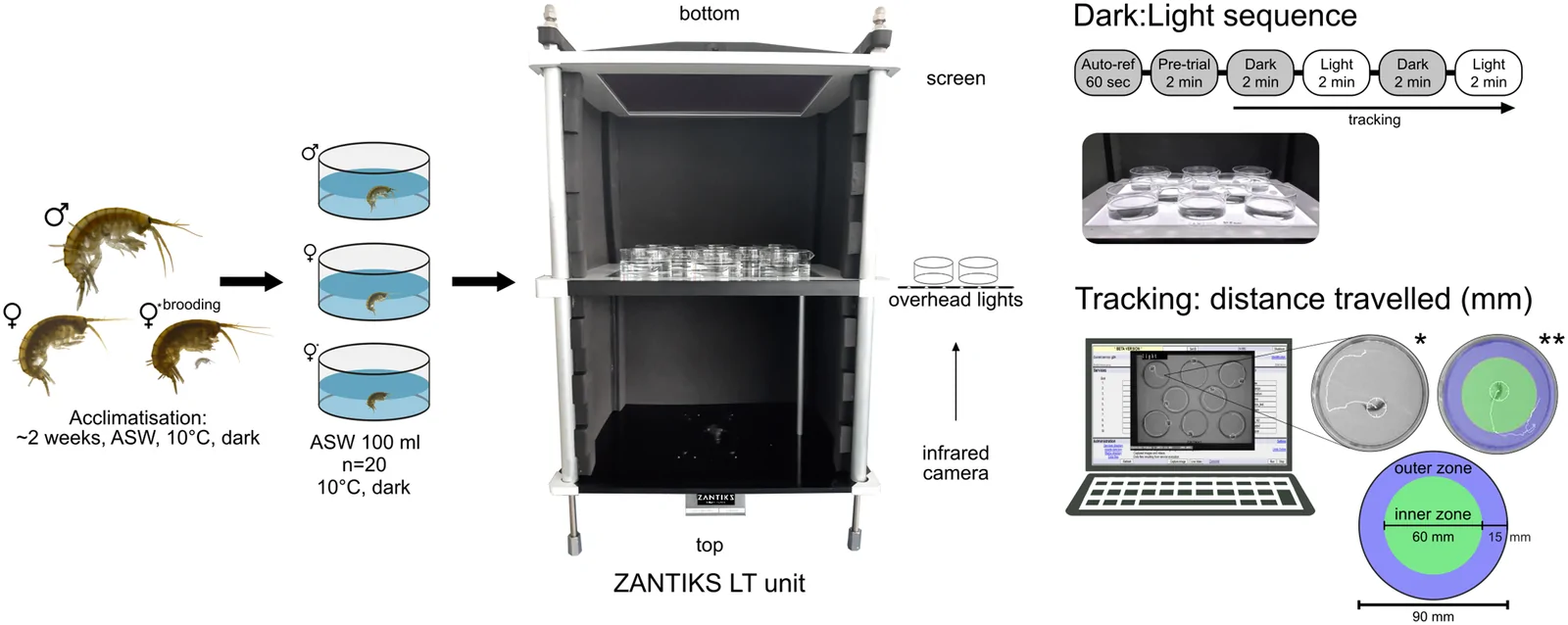
Schematic representation of experimental design of amphipod behavioural assay. Behavioural endpoint as distance travelled (mm) is assessed at an 8-min alternating dark-light conditions using the Zantiks LT system. * 1^st^ behavioural assay all sexes, ** inner and outer zones for 2^nd^ behaviour assay males-only.

### 2.3 Behavioural assessment – water quality oxygen, pH and salinity

A second male-only experiment was conducted with 32 male amphipods (July-collected) to assess potential effects of static incubation in crystallising dishes over 96 h. Behaviour was assessed daily as previously described. Thigmotaxis was quantified using centroid-based tracking by dividing the arena into an inner zone (60 mm diameter) and outer zone (15 mm ring) (Fig 1). Zone assignment was based on centroid position of the animals and reflects spatial preference rather than absolute occupancy. At the start of the experiment, and again daily after behavioural assessments, salinity, dissolved oxygen (DO), and pH were measured in each dish. Temperature of each dish was also recorded after each run. Probes used were: pH (HACH HQ40d multi meter with PHC281 electrode), DO (HACH HQ30s Flexi meter with LDO101 luminescent/optical DO sensor), salinity and temperature (Fisherbrand Traceable Salinity Meter Pen). No moulting events, mortality or effects of dish position were recorded. Males were anaesthetised and photographed as previously described.

### 2.4 Data analysis

Distance travelled (mm) was recorded at 1-second resolution using the Zantiks LT tracking system, which calculates movement based on centroid displacement of each individual. The system automatically tracks animals using built-in object detection, with tracking sensitivity scaled to object size. All recordings were visually inspected to verify correct tracking, and rare tracking errors (<0.02% of data), including temporary loss of detection or misidentification due to shadows or artefacts, were manually corrected (S2 File).

Distances as distance travelled (mm) were summarised over 10-sec, 1-min and 2-min intervals (separated by light conditions), as well as total 8-min intervals for each day (24 h timepoints) and overall (all days pooled). A short 1-sec window (110–130 sec) was used to assess startle responses following the first light switch. Maximum mean speed (mm/s) was averaged per 2-min bin for each sex group and day. Locomotion percentage was derived from frame-to-frame centroid displacement between consecutive seconds. Locomotion was scored as 1 (locomotion) or 0 (no loconmotion), with an intermediate value of 0.5 assigned when individuals occupied both inner and outer zones within a single second, indicating boundary crossing uncertainty.

All statistical analyses were conducted in RStudio v4.3.3 using R (R Core Team, 2024). Principal component analysis (PCA) was performed using prcomp() to examine relationships between distance travelled and dish position.

Locomotion activity (distance travelled) was analysed using linear mixed-effects models (LMMs) fitted with the *lme4* and *lmerTest* packages in R [60,61]. Distance travelled was modelled as a Gaussian response for both the 8-min and 2-min datasets. For the different sex group experiment, Sex group, Day, and Light conditions (Dark1-Light1-Dark2-Light2) were included as fixed effects, including their interactions. Individual identity (ID) was included as a random intercept to account for repeated measurements. For the male-only experiment, Day, Light conditions, and their interaction were included as fixed effects, with ID as a random intercept. Fixed effects were evaluated using Type III analyses of variance with Satterthwaite approximations for denominator degrees of freedom. Model assumptions were assessed using visual inspection of residual and Q–Q plots. Model selection was based on likelihood-ratio tests and Akaike’s Information Criterion (AIC), comparing nested models with alternative fixed-effect structures. Intraclass correlation coefficients (ICC) and marginal and conditional R² values were calculated using the *performance* package [62] to evaluate model fit and variance explained. Estimated marginal means and pairwise comparisons were obtained using the *emmeans* package [63].

To assess the potential influence of body size, body length was included as an additional covariate in the LMMs. For the sex-group experiment, Treatment × body length interactions were additionally evaluated in the 8-min sex group dataset to test whether the relationship between body size and distance travelled differed among groups. Body size effects were further examined using separate linear regression models (Distance ~ body length) within treatment groups. Regression model assumptions were evaluated using diagnostic tests implemented in the *car* package [64].

Group-level comparisons of body length, locomotion percentage, locomotion speed, 10-sec interval data, and water quality parameters were analysed using one-way ANOVAs followed by Tukey HSD post hoc tests. Homogeneity of variances was assessed using Levene’s test; where this assumption was violated, Welch’s ANOVA followed by Games–Howell post hoc tests were applied. Two-group comparisons for 1-sec and 10-sec interval data during light transitions, and for 1-min interval data within sex groups across light phases, were performed using paired t-tests to account for repeated measurements within individual.

Linear regression models were used to examine relationships between female body length and juvenile number, as well as between female and juvenile body length. Linear mixed-effects models were used to test the effects of juvenile number and juvenile size on distance travelled in brooding females over time, with ID included as a random intercept and trial day included as a fixed effect.

## 3. Results

### 3.1 Locomotion assessment in adults of different sex and reproductive conditions

Locomotive behavioural of the male, female, and brooding females were assessed daily over four days across an 8-min period using alternating 2-min dark-light-dark-light conditions using the automated Zantiks LT system (Zantiks Ltd.) (Fig 1).

A general assessment of the three sex groups shows that males were significantly larger than both female groups with 21.3 ± 0.2 mm compared to females with 18.2 ± 0.4 mm (p < 0.001, 95% Cl [2.55, 4.71]) and brooding females with 17.7 ± 0.4 mm (p < 0.001, 95% CI [2.05, 4.20]), respectively. There was no difference in body length between brooding and non-brooding females (p = 0.644, 95% CI [−1.88, 0.87]). All sex groups had similar mean percentages of locomotion over 8-min with locomotion percentage of 59.0 ± 2.6% for males, 61.5 ± 2.7% for females and 61.7 ± 2.6% for brooding females (F(2,53)=0.600, p = 0.552). The LMM of the 8-min data showed that total distance travelled did also not change significantly between sex groups with a total distance of 3917 ± 281 mm for males, 3557 ± 257 mm for females and 3127 ± 203 mm for brooding females (F(2,50)=0.102, p = 0.90), respectively (S3 File). Body length did not significantly explain variation in distance travelled within any sex group, based on separate linear regression models (males: R² = 0.004, F(1,18) = 0.07, p = 0.80; females: R² = 0.019, F(1,15) = 0.30, p = 0.59; brooding females: R² = 0.025, F(1,17) = 0.44, p = 0.51; Fig 2B). Furthermore, the relationship between body length and distance travelled did not differ among sex groups (Sex group * body length interaction: F(2,50) = 0.136, p = 0.873; S3 File). Despite similar mean locomotion values among groups, individual activity levels varied substantially, ranging from 6.5% to 99.6% locomotion and from 263 mm to 11,855 mm total distance travelled per day. Overall, the total distance travelled across all four days constantly decreases with highest distance travelled at the start of the experiment after 24h and a significant decrease after 72h to 96h (Fig 2A and S3 File).

**Fig 2:**
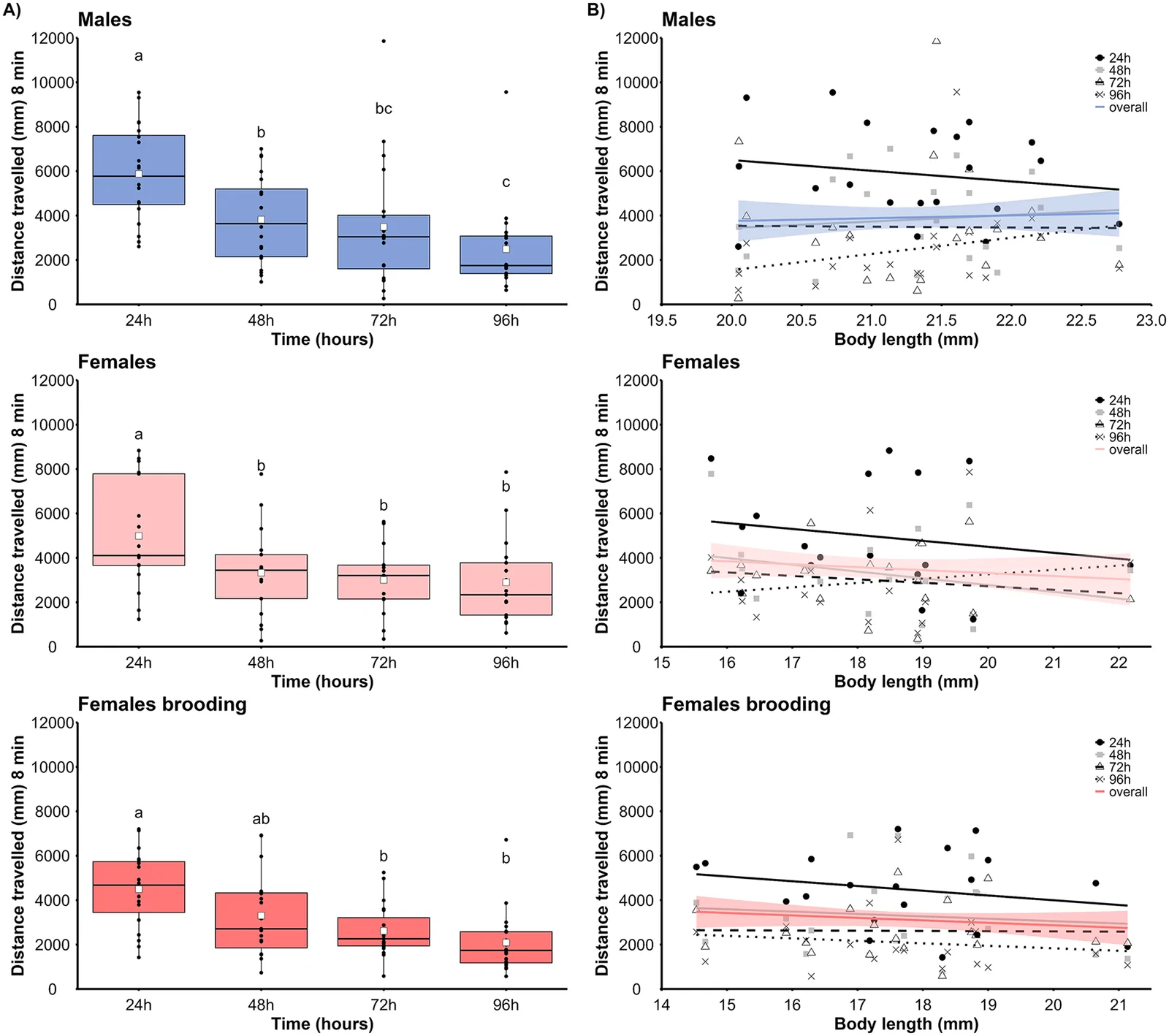
Assessment locomotion behaviour for the three sex groups, males (blue), females (light red) and brooding females (red). **A)** total distance travelled (mm) per day (in hours) per sex group; Black-white squares □ symbolise the mean value. Small letters above plots show significant difference (*p* < 0.05) between days and sex groups. **B)** total distance travelled (mm) plotted along individual amphipod body length. Linear regression lines plotted for distance travelled and amphipod body length for each day (black/grey lines) and overall (coloured lines). Coloured shaded bands denote 95% confidence intervals around the fitted regression lines.

The combined overall data from four days of male, female, and brooding female locomotion show a strong response of all three sex groups to the two 2-min light stimuli during the 8-min assessment (Fig 3A), while locomotion generally decreases during dark periods. The LMM shows a significant difference between all light and dark conditions (Table 1 and S3 File). Across the three sex groups, locomotion during light periods significantly increased compared to the preceding dark period, in particular for male and female amphipods (Fig 3A). Overall, males tend to travel more than both females and brooding females, while brooding females travel the least. This became particularly apparent during the light periods, when males travelled significantly more than brooding females. The 10-sec data also shows that males often travelled significantly more than brooding females during, particularly during the first light phases (Fig 3B). Males also had a significantly higher mean maximum speed during light periods compared to females and brooding females, particularly during the first light trigger (males: 51.8 ± 2.5 m/s significantly faster than females: 41.9 ± 2.4 m/s (p = 0.014, 95% CI[−18.1,-1.65]) and brooding females: 40.5 ± 2.8 m/s (p = 0.09, 95% CI[−20.1,-2.39])), while their maximum speed did not significantly differ during dark periods (S4 File).

**Fig 3:**
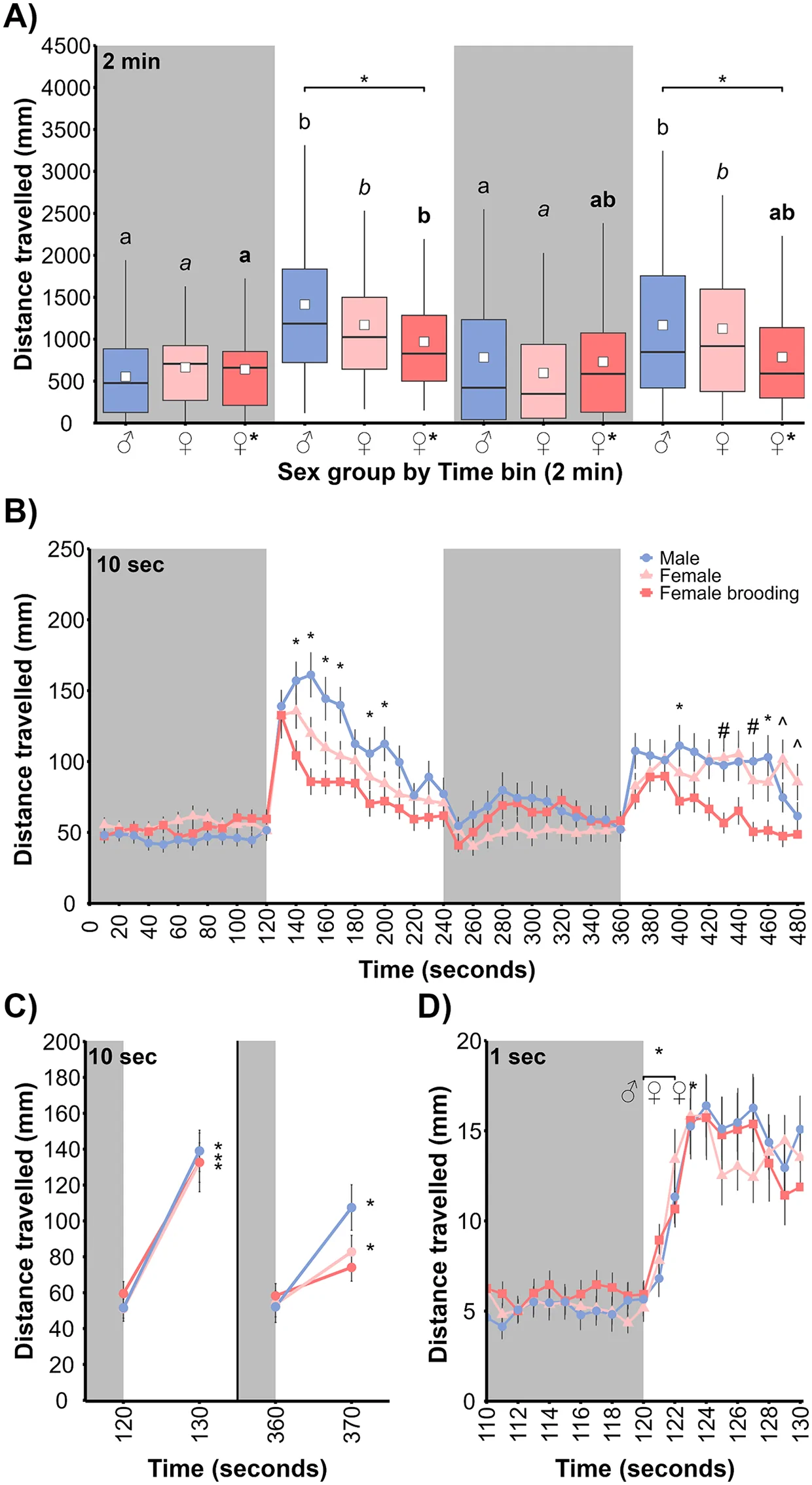
Distance travelled (mm) over 4 days overall for males (♂ blue), females (♀ red) and brooding females (♀* dark red). In **A)** 2-min and **B)** 10-sec time bins for distance travelled (mm). **C)** 10-sec time bin data for both light triggers. **D)** 1-sec time bin data for 1^st^ light trigger. Significant difference p < 0.05 with * between males & br. females, # br. females to males & females, ^ between females & br. females, small letters above plots between light conditions for each sex group, * between sex groups (2-min data) or between time points (1-sec & 10-sec light switch). Black-white squares □ symbolise the mean value.

**Table 1:** Output for Linear Mixed Model (LMM) of 2-min distance travelled data between different fixed effects: sex groups (Sex), Days and Dark1-Light1-Dark2-Light2 (Light conditions) across all four days (overall), with body length as covariant. Significance level p <  0.05*, <  0.01**, <  0.001***.

COMPARISON	Num df	Den df	F	p-value	
Sex	2	52	1.466	0.24	
Days	3	795	45.755	<0.001	***
Light conditions	3	795	40.613	<0.001	***
BodyLength	1	52	0.473	0.495	
Sex:Days	6	795	1.211	0.298	
Sex: Light conditions	6	795	4.325	<0.001	***
Days: Light conditions	9	795	0.572	0.82	
Sex:Days: Light conditions	18	795	0.789	0.715	
ICC = 0.215					

The strongest change in locomotion behaviour for all three sex groups occurred during the first 10 seconds after the first light trigger (male: t(136)= −6.35, p < 0.001; female: t(97)= −6.67, p < 0.001; female brooding: t(99)=−4.13, p < 0.001) (Fig 3C). A closer look reveals that this response occurs during the first 3 seconds after the first light trigger at 120 seconds (Fig 3D) (male: t(131)= −5.26, p < 0.001; female: t(99)= −6.28, p < 0.001; female brooding: t(94)= −4.23, p < 0.001). For the second light trigger, only males and females significantly increased their locomotion (male: t(140)= −3.60, p < 0.001; female: t(134)= −2.51, p < 0.001) (Fig 3C). This can also be observed in the 10-sec data (Fig 3B), where the first light trigger appears to have a stronger effect during the first 60 sec, while the second light response seems weaker and much broader. This is supported by the 1-min data, which showed a general stronger effect during the first 60 sec of the first light trigger than during the second light trigger (S4 File).

The data were also assessed for each individual day. Significant fluctuations in locomotion behaviour in animals can be observed across the daily assessments and light triggers, regardless of the animal’s sex (Fig 4 and S3 and S4 Files). On each assessment day, the first light trigger led to a consistent significant spike in locomotion during the first 10 seconds (2–4 seconds in 1-sec time bins) in each sex group. The second light trigger varied in its effectiveness in significantly increasing locomotion. On day two (48h) only males and females, and on day four (96h) only brooding females showed a significant increase in locomotion within the first 10 seconds. Overall, the individual 10-sec day data highlights the variability between days in which on the first assessment day animals seemed more active compared to the last day. No significant difference in total distance travelled between sex groups per day has been observed (S3 & S4 File).

**Fig 4:**
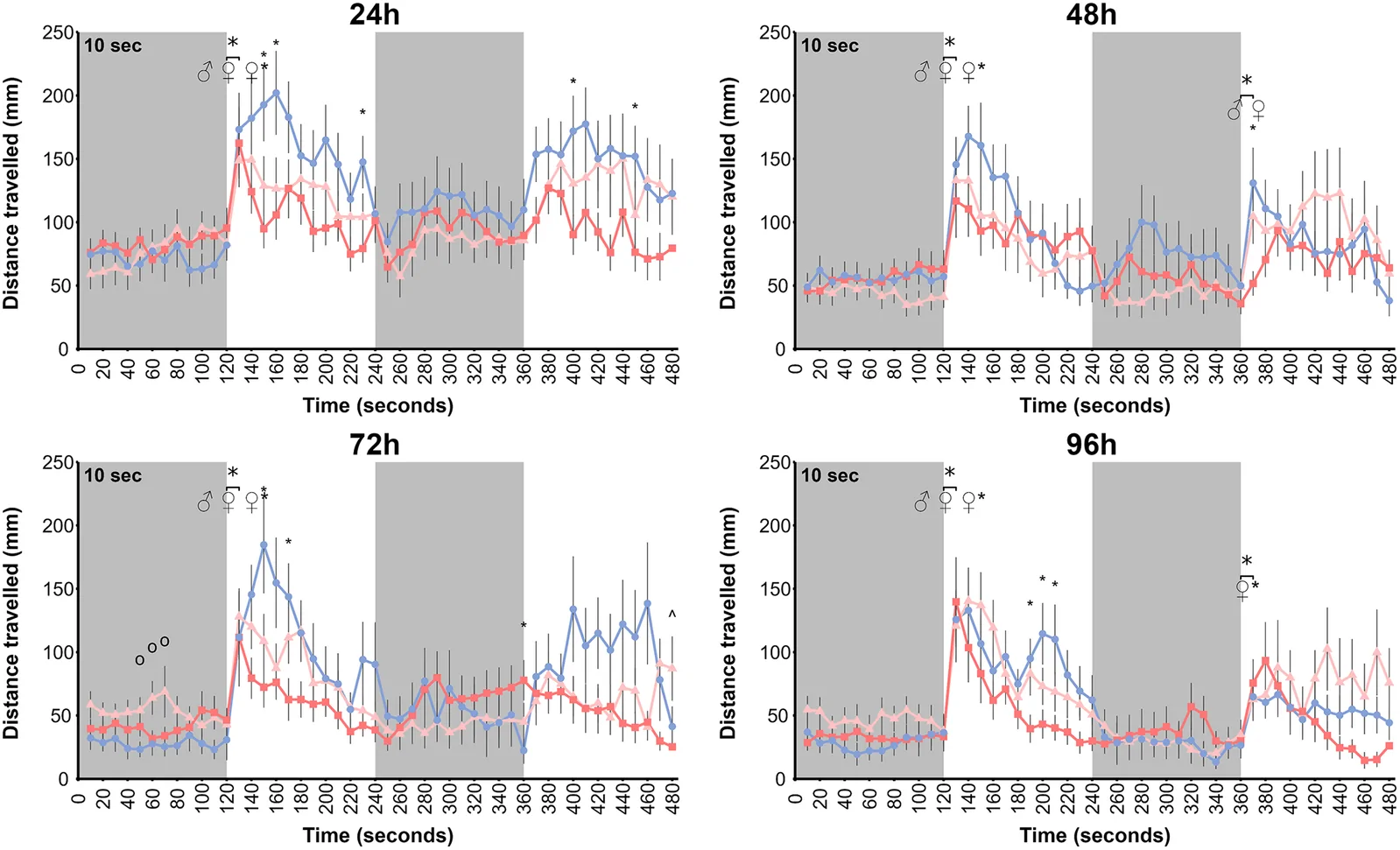
Distance travelled (mm) of amphipods for individual days. In 10-sec time bins for males (♂ blue), females (♀ red) and brooding females (♀* dark red). Significant difference p < 0.05 with * between males & br. females, ^ between females & br. females, ○ females & males. * significant difference between 120/130 seconds and 360/370 seconds.

To test if the lower overall distance travelled in brooding females is related to them carrying brood, juveniles released and remaining in the marsupium during assessment were counted and measured (Fig 5). The number of juveniles can vary from 4 to 33 individuals per brooding female. Body length of juveniles remaining in marsupial and released range from 2.27 mm to 5.09 mm. Larger females, although not significant, tend to carry more juveniles, while also being only marginally bigger (number: R² = 0.122, F(1,20)=2.79, p = 0.11; body length: R² = 0.082, F(1,20)=1.78, p = 0.196) (Fig 5A). Brooding females did not release all juveniles at once, but had several release events (up to three events during 96h assessment), during which they released one to seven juveniles per release event. Six of the twenty-two brooding females did not release any juveniles (number of juveniles in marsupium ranged from 4 to 25 per female), and one female released all juveniles (initially eight juveniles). There was no significant difference between the body length of released and remaining juveniles in the marsupium with the mean body length in marsupium of 3.24 ± 0.03 mm and released of 3.16 ± 0.04 mm, respective (t(189)= 1.676, p = 0.095) (Fig 5B). Neither number of juveniles nor juvenile body length had a significant effect on the total distance travelled over time in brooding females (number juveniles: F(1,27)=0.006, p = 0.938); body length: F(1,27)=0.062, p = 0.806; Fig 5C and S3 File).

**Fig 5:**
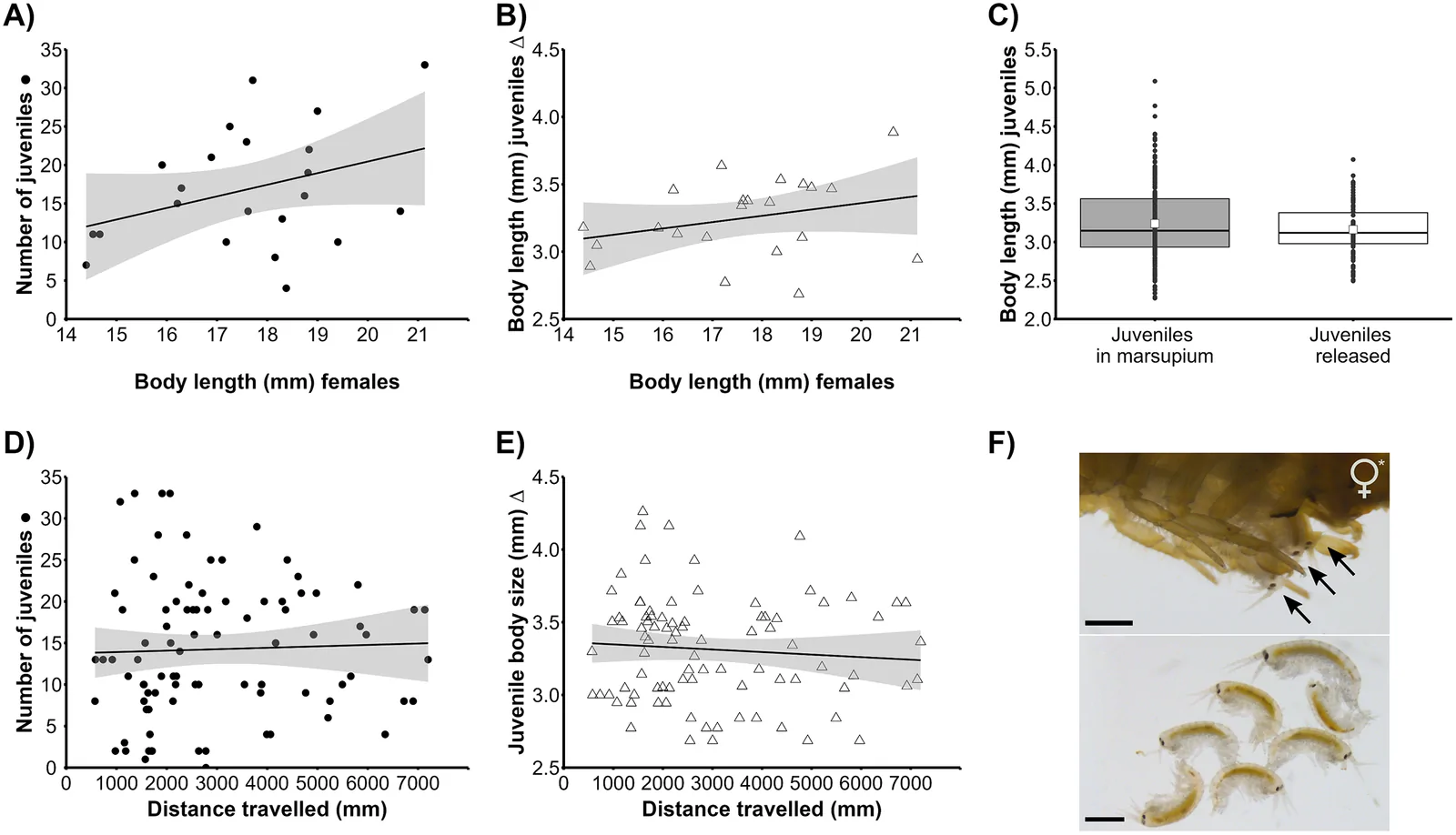
Brooding female and juvenile assessment. Relationships between female body length (mm) and **A)** number of juveniles; and **B)** body length (mm) of juveniles; **C)** body length (mm) of juveniles in marsupium and released. Relationships between female distance travelled (mm) over 8-min and **D)** number of juveniles in marsupium, and **E)** body length (mm) of juveniles; **F)** juveniles (black arrows) present in female marsupium and after release. Scale bar: 1 mm. Linear regression lines plotted for plots **A**, **B**, **D** & **E**. Shaded bands denote 95% confidence intervals around the fitted regression lines.

### 3.2 Locomotion assessment adults – water quality

A second experiment with male-only animals was conducted to test for specific wall-hugging behaviour as well as any changes in water quality (Fig 1). Males were selected for this experiment as they showed the highest overall locomotive activity and the strongest and most consistent response to light stimuli in the sex group experiment. Amphipods of the male-only experiment displayed similar behaviour as observed in the first experiment, with a few notable differences. No significant decline in overall distance travelled during the 8-min assessment across the four days was observed (F(3,93)=1.44, p = 0.235; Fig 6A) as was seen in the first experiment. On the first day (24h), the males in the male-only experiment travelled significantly less on average (male-only experiment: 3975.1 ± 448.0 mm) than the males on the first day of the first experiment (sex experiment: 5876.0 ± 476.7 mm, Fig 2A) (t(47) = 2.01, p = 0.0056), but there was no difference in the total distance travelled on subsequent days.

**Fig 6:**
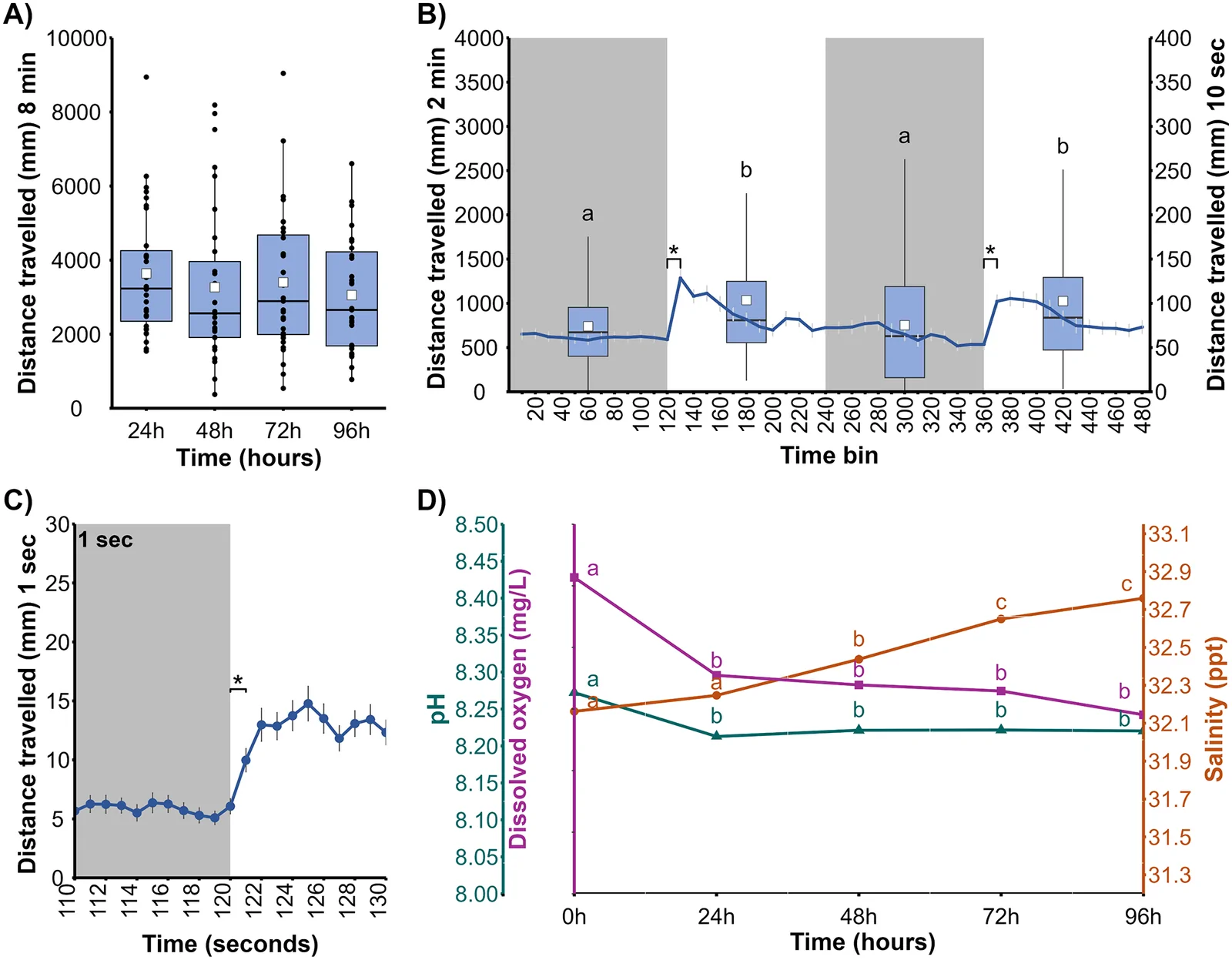
Distance travelled (mm) over 4 days overall for 32 male amphipods. **A)** in 8-min data for each individual day, **B)** 10-sec and 2-min time bins combined for overall data, and **C)** in 1-sec time bin for first light switch of overall data. **D)** Mean salinity (ppt), dissolved oxygen (mg/L) and pH for all dishes with animals assessed daily including at the start of the experiment. Black-white squares □ symbolise the mean value. * Significant difference for 10-sec & 1-sec data. Small letters symbolise significance between light conditions or days.

Similar to the sex experiment, in the male-only experiment, the LMM shows that male amphipods’ swimming behaviour was significantly stimulated by both light triggers (Fig 6B and S3 File). A strong light response was recorded during the first 2 seconds after the first light switch, with the first significant increase in distance travelled occurring at 121 seconds (t(178)= −4.50, p < 0.001; Fig 6C). During each assessment, locomotion behaviour in relation to the inner and outer zones of the dish was also evaluated. On average, the amphipods spent 63.8 ± 1.7% of their time swimming (locomotion) and 87.6 ± 1.1% of their time in the outer zone. Of their total locomotion, 90.6 ± 0.7% occurred in the outer zone. The percentage of locomotion or time spent in the outer zone did not vary between days (locomotion: F(1,126)=2.17, p = 0.143; outer zone: F(1,126)=0.17, p = 0.679) (S5 File). In addition, body length was included as a covariate in the 8-min LMM to assess whether individual size influenced locomotor activity. Body length did not significantly affect total distance travelled during the assessment (F(1,30) = 1.58, p = 0.22; S3 File). A weak decreasing trend between body length and distance travelled was observed in a separate regression analysis, but this relationship was not significant (R² = 0.05, F(1,30) = 1.581, p = 0.218). pH and DO levels dropped significantly within the first 24 h, from pH 8.27 ± 0.003 and DO 10.03 ± 0.03 mg/L to pH 8.21 ± 0.002 (t(62)=18.71, p < 0.001) and DO 9.71 ± 0.02 mg/L (t(54)=9.95, p < 0.001), respectively (Fig 6D). After this initial decrease, pH and DO levels remained stable for the remaining 72 h. Salinity, however, increased significantly over the course of the experiment, from 32.16 ± 0.01 ppt to 32.76 ± 0.04 ppt (t(37)= −13.89, p < 0.001). This increase may have been caused by weak evaporation of seawater from the dishes. A gradual increase in temperature of 4.6 ± 0.1°C was recorded across crystallising dishes over the course of each 11-min recording period.

## 4. Discussion

### 4.1 Behavioural assay and light triggers

High-throughput approaches are key to accurately assessing behavioural traits in animals across a large number of replicates. The number of animals that can be tested in a short period often depends on the chosen experimental apparatus, the behavioural endpoints assessed, the species, animal size, required experimental vessel size, and the number of treatments We successfully tracked eight *M. marinus* amphipod individuals for an 8-min period without significant mistracking or manual corrections using the automated Zantiks LT system. Individuals showed a rapid increase in locomotive activity immediately following light onset across all sexes, consistent with previously reported light-induced behavioural responses in *M. marinus* and other amphipod species using different experimental setups [31,38–43,47,48]. The magnitude and consistency of this response varied across sex groups and days, with males showing the most pronounced and repeatable response to light transitions.

A rapid behavioural response within seconds of light onset, particularly after the first light switch, was consistently observed, indicating a strong startle-like reaction to sudden changes in illumination. This response has been described in other amphipod species as an acute escape reaction to abrupt environmental stimuli, although the temporal dynamics appear to vary among taxa. While *M. marinus* displayed immediate swimming activity following light onset (this study and [48]), *Gammarus pulex* has been reported to exhibit a brief freezing phase prior to locomotive activation [43]. These differences highlight potential species-specific variation in the expression of rapid escape behaviours and may be relevant for comparative ecotoxicological assessments.

Repeated light triggers often led to a weaker response to the second light pulse in *M. marinus* (as seen in this study and others [31,38–43,47,48], though effects were temporary and disappeared by the following day. This may result from overstimulation of the amphipods’ optic nerve [42]. High-intensity light can damage crustacean retinas ([65] and references therein), so experimental light intensity must balance eliciting measurable responses with avoiding harm. Previous research on *M. marinus* and *G. pulex* have shown that, although amphipods react to light triggers (phototactic response) regardless of different light intensities (200, 800, 2000, 4000 lux), the mean velocity increased with increasing light intensity [31]. Hence, differences in the intensity of a behaviour might change with light intensity. For *M.* *marinus*, 200–800 lux appears acceptable (~ 300 lux was used in this study) and closer to their natural habitat (estimated approx. 500−1,000 lux at 1 meter in UK estuaries [66]).

### 4.2 Variation between sex groups

Behavioural differences between sexes are an important consideration, as endpoints such as swimming may be influenced by sex-specific morphology and behaviour. In the current study, *M. marinus* males did not swim significantly more than females or brooding females over the entire 8-min experiments. However, when assessed in 10-sec and 2-min time bins, differences between sexes could be observed. These differences generally showed that: (a) males moved significantly more than females, and especially brooding females, during the light phases; (b) significant locomotion changes in males between dark and light phases were more consistent across all days; and (c) the increase in locomotion of males within the first 10 seconds after a light trigger was stronger and more consistent across days. Overall, males showed the strongest light response, followed by females, with brooding females responding least. These findings contrast with previous work reporting higher female swimming speeds during dark phases and similar velocities during light phases [39]. In our study showed, males displayed higher mean maximum speeds across all dark and light phases, and were significantly faster during both light phases. Differences in arena size may partly explain this discrepancy. The previous study used smaller dishes (36 mm in diameter), whereas we used a larger dishes (90 mm in diameter), may have reduced potential “ceiling” effects limiting male performance [39]. Higher velocity in males, compared to females and brooding females, has also been observed in the freshwater amphipod *Gammarus pseudoliminaeus* [67]. Although *M. marinus* males were significantly larger than both female groups in the present study, body length was not significantly associated with distance travelled within males, females, or brooding females, and inclusion of body length as a covariate did not improve the mixed-effects models. Therefore, we found no evidence that body length influenced distance travelled in our dataset. Previous studies in herbivorous freshwater amphipods have reported positive relationships between body length and locomotive performance [29], suggesting that size may contribute to locomotion under some conditions. Furthermore, in our study *M. marinus* individuals were not selected according to body length, resulting in limited overlap in body length distributions among sex groups (1 female, 2 brooding females, 17 males, body length range 20–22 mm). Consequently, the relative contributions of sex and body length could not be fully disentangled in the present study. Future studies using size-matched males and females would be valuable for separating the effects of morphology and sex on locomotive behaviour. The choice of using one or both sexes in exposure experiments should depend on the compound’s mode of action (impact on male and/or female physiology and behaviour) and the desired sensitivity, as males may provide a stronger light response. Regardless, sex, body length, and brooding status should be recorded and considered during experimental design and analysis, as these factors may influence behavioural responses and could otherwise confound treatment effects.

Brooding females might pose particular challenges in exposure experiments, as they carry juveniles in their marsupium, and the number of juveniles varies greatly over time and between females [68]. While brood number did not correlate with distance travelled, brooding females showed consistently weaker responses to light compared to non-brooding females and males. Carrying a brood, irrespective of size, may therefore reduce responsiveness to light stimuli, potentially due to increased energetic constraints or reduced locomotive flexibility associated with brood carriage.

Seasonal variation may influence behavioural responses. Males collected in January (mean seawater temperature 9.4 °C) travelled greater total distances on day one than males collected in July (18.5 °C). Although both groups were acclimated at 10 °C for two weeks prior to testing, individuals originating from warmer summer conditions may have reduced activity under colder incubation temperatures, but future work should examine how seasonally relevant incubation temperatures and light regimes influence responses.

### 4.3 Daily variations and experimental conditions

A significant decrease in overall locomotion was observed across all sexes, indicating behavioural variation between days. While light stimuli consistently triggered a startle response with increased locomotion over all four days, animals were generally less active on the subsequent day, resulting in a decrease in total distance travelled. These daily variations may reflect habituation as previously suggested for *M. marinus* [41]. Habituation, which is a change in baseline behaviour due to the disappearing of initial stress or novelty of the environment, is a commonly reported in repeated fish assays [69,70]. While some amphipod studies recommend replacing animals daily [32,71], this is often impractical in long-term exposure experiments. Where feasible, pre-habituation to the experimental vessel prior starting the behavioural assessment may reduce variability. As animals are not fed during experiments, starvation effects are a consideration, though they are unlikely here. Freshwater amphipods showed that non feeding up to 10 days does not affect the swimming activities of amphipods [72], and marine amphipods showed less chaotic exploratory behaviour in animals starved for 24 h compared to non-starved [73]. Feeding during exposure studies is often impractical, as test compounds may bind to food and introduce additional variability. Survival rates in *M. marinus* and *G. pulex* seem not to be affected by short-term starvation periods, and non-feeding protocols have been successfully used for up to 21 days [30,42,47,48]. Despite controlled conditions, substantial variation persisted across days and individuals. Some amphipods remained nearly motionless one day but became highly active swimmers the next, with activity ranging from minimal movement to near-continuous swimming. In addition, inter-individual differences were evident across repeated measurements, with some individuals consistently showing higher or lower activity levels than others. Such variation can complicate data interpretation, and inclusion of intermediate assessment days, rather than only baseline and endpoint measurements, is advisable to avoid misinterpretation due to an anomalously low-activity day that is not linked to any treatments. However, daily measurements should be applied cautiously, as they may contribute to habituation.

To ensure comparability with previous studies on *M. marinus* [31,39–41,48], animals were acclimated under constant darkness. Continuous darkness has been shown to suppress circadian and circatidal rhythms in this species after seven days [58], thereby reducing rhythmic variability that may otherwise persist in laboratory settings despite the absence of tidal cues. While this approach improves experimental standardisation, it does not reflect the natural light environment of this intertidal amphipod and may be questioned in ecotoxicological contexts favouring environmentally realistic conditions. Currently, no guidelines define optimal acclimation light regimes for *M. marinus*, and further work is needed to evaluate the trade-off between minimising endogenous rhythmicity and maintaining ecological relevance, particularly regarding responsiveness to light stimuli and potential seasonal influences. Recent work on artificial light at night reported reduced activity, rhythmicity, and survival under constant darkness in *M. marinus* [74], although light-triggered responses were not examined. In contrast, mortality remained low in the present study. Evidence from *G. pulex* indicates that reliable light-induced responses can be obtained under varying acclimation regimes [31,43,47], yet this species appears less sensitive to nocturnal light pollution than *M. marinus* [74], suggesting possible species-specific effects. Determining optimal acclimation conditions therefore remains an important area for future behavioural standardisation.

The choice of arena size represents a trade-off between maximising throughput and providing sufficient space to detect behavioural differences. Although the dish size used here was appropriate for the experimental setup, distance travelled by larger individuals was slightly reduced, suggesting that larger arenas may be beneficial. Larger animals occupy more space and may move faster, reaching arena boundaries more quickly. In freshwater amphipods, body length generally correlates with higher velocity [29]. Previous work on *M. marinus* and *Artemia* has shown that larger arenas yield higher recorded activity [40,75]. In contrast, overly small arenas may impose a “ceiling” effect, limiting behavioural expression and potentially leading to false negatives.

Dish size may also influence thigmotaxis, a behavioural endpoint used in previous studies. Thigmotaxis, or “wall-hugging” behaviour, describes the tendency of animals to remain close to boundaries and is widely applied as an anxiety-related endpoint in rodents [76] and zebrafish [77,78]. Although wall-oriented behaviour has been reported in terrestrial amphipods [79] and may occur in aquatic species, not all boundary-associated movement necessarily reflects thigmotaxis. In the present study, amphipods spent considerable time near the outer zone of the dish. However, individuals did not consistently orient toward or actively seek walls after ceasing movement, and boundary proximity often coincided with arena limits during light exposure. These observations suggest that wall-associated behaviour in small dish-based arenas may partly reflect spatial constraints rather than deliberate boundary preference. Thigmotaxis should therefore be interpreted cautiously in confined circular setups, and alternative designs such as larger mazes [80] or semi-field systems [47] may provide more suitable conditions for assessing this endpoint.

Dish size also determines the volume of water in which the amphipods are immersed, potentially affecting water quality over time and influencing how frequently water should be changed in static systems. *M. marinus* lives in estuarine and coastal environments and tolerates short-term fluctuations in salinity, pH, and dissolved oxygen (DO), although growth and reproduction may be affected under suboptimal conditions. For example, pH below 7.5 and salinity below 22 ppt have been shown to negatively affect embryonic development in *M. marinus* [81]. In the marine amphipod *Melita longidactyla* moderately low oxygen levels (3.5–4.5 mg/L O2) impair growth, respiration, and reproduction after 3–4 weeks [82], a trend also observed in freshwater amphipods [83,84]. Oxygen levels below 3.5 mg/L O2 can also disrupt copulation behaviour leading to separation of copulating pairs or failure to copulate altogether [82,85]. In our study, although changes in water quality were statistically significant, they did not approach previously reported harmful thresholds. The initial drop in DO and pH after 24 h likely resulted from introducing animals into the dishes rather than sustained oxygen depletion, as DO declined only gradually over the following 96 h. The increase in salinity (≈0.5 ppt) was most likely due to slow evaporation, as dishes were loosely covered to allow gas exchange. Determining water-change frequency in static systems requires balancing optimal water quality with minimising disturbance and chemical use. The 96-hour period applied here appears suitable for *M. marinus*, consistent with previous static behavioural assays recommending water renewal every two to four days [30,42,44,47,48], provided chemical stability is ensured. Unlike other commercially available systems (e.g., DanioVision and ToxmateLab), the Zantiks observation chamber does not provide active temperature control. A gradual increase in temperature during recordings therefore cannot be excluded. While *M. marinus* naturally experiences fluctuating intertidal temperatures, and is therefore likely tolerant to short-term thermal variation, the potential influence of short-term warming on locomotive activity cannot be fully ruled out. Future studies would benefit from improved temperature stabilisation or continuous within-dish monitoring to further reduce this potential source of variation in behavioural endpoints.

## 5. Conclusion

In conclusion, the automated assessment of *M. marinus* locomotion using the Zantiks LT system proved to be a reliable approach, revealing robust sex- and light-dependent behavioural patterns. Males exhibited the strongest phototactic response, while brooding females showed reduced activity, regardless of brood size. These findings emphasize the importance of considering sex, body length, and environmental conditions in behavioural assays. The results also highlight the value of repeated measurements, pre-habituation, and careful arena design to minimize confounding variability. Despite daily variation and potential habituation effects, light remained a reliable inducer of locomotion, particularly during the first few seconds post-stimulus. Biological factors, like body length and brood load, might influenced behavioural responses and must be considered in experimental design. A special focus should also be placed on potential variations in behaviour due to seasonal changes. Arena size emerged as a potential limiting factor for larger individuals, underscoring the need to match the experimental setup with organism characteristics to avoid underestimating behavioural responses. Overall, critical parameters must be tailored to the specifics of the species, the research question, and the overall scope of the project. When using *M. marinus* as an ecotoxicological model in behavioural assays, several key factors remain to be addressed, including the effects of incubation temperature and light regime. Additionally, direct comparisons between different observation chambers would help identify subtle differences in the success of recording locomotion responses across various tracking systems.

Nonetheless, pairing locomotion with a light stimulus consistently elicits a strong and quantifiable behavioural response for this group of crustaceans across a range of high-throughput systems like the Zantiks unit (used in this study), and previous studies using DanioVision, ToxmateLab, and other commercial and non-commercial recording units. This consistency across different systems will facilitate the development of standardized guidelines for ecotoxicological assessments across laboratories, regardless of the monitoring equipment used. The choice of which unit to purchase or whether to opt for a home-made solution often depends on factors such as financial resources, availability, and whether the setup is used for different species (e.g., fish, rodents). Amenities like controlled temperature, water flow, or automatic chemical dispersion often come with higher costs, which may not be feasible for laboratories with limited financial resources. Therefore, standardized guidelines for behavioural assays should be designed with the feasibility and availability for most laboratories in mind.

## Supporting information

S1 File Dish position and PCA. (DOCX)

S2 File Tracking issues. (DOCX)

S3 File Linear mixed models. (XLSX)

S4 File Locomotion behaviour amphipods sex groups. (DOCX)

S5 File Locomotion behaviour amphipods male-only. (DOCX)
