# Supplementary material for: Shedding light on amphipod behaviour: Baseline locomotion behaviour for laboratory ecotoxicology experiments
Source: PLoS One. 2026 Oct 7;21(10):e0357763. doi: 10.1371/journal.pone.0357763 (PMC13645043; doi:10.1371/journal.pone.0357763)
Supplement: Supplementary file 1 [file pone.0357763.s001.docx]

**S1 File:** **Dish position and PCA**

**Figure S1: Dish position and principal component analysis (PCA). A)** Schematic representation of dish positions when placed in the Zantiks observation unit. **B)** Example of dish positions in a tracking video from the Zantiks unit. The positions appear mirrored due to the observation unit being placed upside down. **C)** PCA of sex group experiment, with different colours representing the different groups and letters indicating the dish positions within observation unit. **D)** PCA of male-only experiment with different colours showing the different dish positions for each amphipod.
