# Supplementary material for: Shedding light on amphipod behaviour: Baseline locomotion behaviour for laboratory ecotoxicology experiments
Source: PLoS One. 2026 Oct 7;21(10):e0357763. doi: 10.1371/journal.pone.0357763 (PMC13645043; doi:10.1371/journal.pone.0357763)
Supplement: Supplementary file 2 [file pone.0357763.s002.docx]

**S2 File:** **Tracking issues**

Tracking issues included loss of tracking of the animal or sudden mis-tracking due to another object (e.g., remains of faeces or from juvenile release) or shadow (Figure S2, Table S2). When tracking issues occurred, data were manually corrected as follows:

1. **Initial non-tracking of the animal at the start of assessment:** Occasional non-detection of the animal occurred when the animal was not moving initially, resulting in either no tracking or random tracking of the dish itself (Figure S2A). After confirmation of non-movement by manual inspection, the distance travelled was set to zero until the animal started moving. In all cases, the software started tracking the animal as soon as the animal started moving.
2. **Mis-tracking due to another object or shadow:** Sudden mis-tracking by the software (Figure S2B) led to incorrectly high distances recorded, with three options for correction:
   a. When tracking was disrupted during locomotion for less than 2 seconds, a mean distance travelled was calculated for this time point based on the distance travelled 2 seconds before and after the tracking issue.

**Justification:** This case only happened when animals were in constant locomotion, usually swimming in circles in the outer zone (along the dish edge), and manual inspection showed no visible change in speed, assuming constant locomotion.

b. When tracking issues occurred for longer periods during locomotion, speed consistency was more difficult to predict, and this data point should therefore be excluded from the dataset.

**Note:** No such case was observed in any of the experiments, but it occurred very rarely during the initial testing phase.

c. When tracking issues occurred with non-moving animals during assessment (not from the beginning), the distance travelled was set to zero until the animal started moving.

**Note:** No such case was observed in any of the experiments, but it occurred very rarely during the initial testing phase.

**Figure S2:** Examples non-tracking (A) and mis-tracking (B) during assessment with blue arrow showing non-tracked animals and red arrow shows mis-tracking.

| **Table S2**: Tracking issues summary across both experiments with a total of 176,640 measurement points taken across both behavioural experiments (92 animals). | | | |
| --- | --- | --- | --- |
|  | **Non-tracking initially** | **Mis-tracking locomotion <2sec** | **% tracking issue total** |
| **Measurement points corrected total** | 32 | 3 | 0.020% |
| **Occurrence** | 1 | 3 |  |
| **Animals involved** | 1 | 2 |  |
