# Supplementary material for: Shedding light on amphipod behaviour: Baseline locomotion behaviour for laboratory ecotoxicology experiments
Source: PLoS One. 2026 Oct 7;21(10):e0357763. doi: 10.1371/journal.pone.0357763 (PMC13645043; doi:10.1371/journal.pone.0357763)
Supplement: Supplementary file 3 [file pone.0357763.s004.docx]

**S4 File:** **Locomotion behaviour amphipods sex groups**

**Figure S4A: Overall percentage (%) of time spend in locomotion per day per sex group.** Each day for males (♂ blue), females (♀ red) and brooding females (♀* dark red). Significant difference p<0.05 * between days marked with small letters.

**Figure S4B: Mean maximum speed (mm/s) per day per sex group in 2-min time bins** for males (♂ blue), females (♀ red) and brooding females (♀* dark red) for overall and each day (24h ●, 48h ∆, 72h ■, 96h ×). Significant difference p<0.05 * between sex groups within light conditions.

**Figure S4C: Distance travelled (mm) of amphipods in 1-min time bins** for males (♂ blue), females (♀ red) and brooding females (♀* dark red) for overall data. Significant difference p<0.05 with * between sex groups within light conditions. Black-white squares □ symbolise the mean value.

**Figure S4D: Distance travelled (mm) of amphipods for individual days.** In 2-min & 8-min time bins, 10-sec for both light switches and 1-sec time bin for the 1^st^ light switch for males (♂ blue), females (♀ red) and brooding females (♀* dark red) for each individual day. Significant difference p<0.05 indicated as small letters above bars between light conditions for each sex group, * between sex groups within light conditions and 1-sec time bins, and * between 10-sec light conditions. Black-white squares □ symbolise the mean value.
