# Supplementary material for: Shedding light on amphipod behaviour: Baseline locomotion behaviour for laboratory ecotoxicology experiments
Source: PLoS One. 2026 Oct 7;21(10):e0357763. doi: 10.1371/journal.pone.0357763 (PMC13645043; doi:10.1371/journal.pone.0357763)
Supplement: Supplementary file 4 [file pone.0357763.s005.docx]

**S5 File:** **Locomotion behaviour amphipods male-only**

**Figure S5: Behaviour of male-only experiment for individual days. A)** Overall percentage time spend in locomotion, and in outer zone during 8-min including representation of amphipod swimming in outer zone; **B)** Scatter plot of total distance travelled (mm) plotted along individual amphipod body length. Coloured shaded bands denote 95% confidence intervals around the fitted regression lines; **C)** Distance travelled in 10-sec time bins combined with 2-min time bins for male amphipods for each individual day including distance travelled in inner zone (dark blue) and outer zone (light blue). Significant difference p<0.05 with small letters above bars between light conditions. Black-white squares □ symbolise the mean value.
